# Association between low-dose pulsed intravenous cyclophosphamide therapy and amenorrhea in patients with systemic lupus erythematosus: A case-control study

**DOI:** 10.1186/1472-6874-11-28

**Published:** 2011-06-10

**Authors:** Sayumi Baba, Yasuhiro Katsumata, Yasushi Kawaguchi, Takahisa Gono, Tomoko Sugiura, Tokiko Kanno, Masako Hara, Hisashi Yamanaka

**Affiliations:** 1 Institute of Rheumatology, Tokyo Women's Medical University, 10-22 Kawada-cho, Shinjuku-ku, Tokyo 162-0054, Japan

## Abstract

**Background:**

The risk for amenorrhea following treatment of systemic lupus erythematosus (SLE) patients with low-dose intravenous cyclophosphamide (IVCY) has not been fully explored. Our objective was to ascertain the incidence of amenorrhea following treatment with low-dose IVCY and the association between amenorrhea and the clinical parameters of SLE.

**Methods:**

A case-control retrospective study of premenopausal women ≤ 45 years old who had been treated for SLE with low-dose IVCY (500 mg/body/pulse) plus high-dose glucocorticoids (0.8-1.0 mg/kg/day of prednisolone; IVCY group) or glucocorticoids alone (0.8-1.0 mg/kg/day of prednisolone; steroid group) in our hospital from 2000 through 2009 was conducted using a questionnaire survey and medical record review.

**Results:**

Twenty-nine subjects in the IVCY group and 33 subjects in the steroid group returned the questionnaire. A multivariate analysis revealed that age at initiation of treatment ≥ 40 years old was significantly associated with amenorrhea [*p *= 0.009; odds ratio (OR) 10.2; 95% confidence interval (CI) 1.8-58.7]. IVCY treatment may display a trend for association with amenorrhea (*p *= 0.07; OR 2.9; 95% CI 0.9-9.4). Sustained amenorrhea developed in 4 subjects in the IVCY group and 1 subject in the steroid group; all of these patients were ≥ 40 years old. Menses resumed in all subjects < 40 years old, irrespective of treatment.

**Conclusions:**

Although low-dose IVCY may increase the risk for amenorrhea, our data suggest that patients < 40 years old have a minimum risk for sustained amenorrhea with low-dose IVCY treatment. A higher risk for sustained amenorrhea following treatment with IVCY is a consideration for patients ≥ 40 years old.

## Background

Systemic lupus erythematosus (SLE) remains a therapeutic challenge. One of the most successful therapies for severe SLE has been administration of monthly intravenous cyclophosphamide (IVCY) for 6 months followed by quarterly maintenance infusions for 2 years (traditional/NIH IVCY regimen) [1-3]. However, the side effects of long-term exposure to cyclophosphamide include infection, bone marrow damage, malignancy, hemorrhagic cystitis, and ovarian dysfunction [2]. Ovarian dysfunction may result in physiological changes associated with menopause, including loss of bone mineral density, hypercholesterolemia, onset of vasomotor and genitourinary symptoms, psychological distress, weight gain, and infertility [4]. Because SLE mainly affects premenopausal women, ovarian dysfunction is a particularly serious adverse event in SLE and may be particularly distressing for women who have not completed their family.

The incidence of cyclophosphamide-induced amenorrhea is generally related to age and cumulative dosage of cyclophosphamide [4]. Although most data for understanding the biological characteristics of cyclophosphamide gonadotoxicity comes from the oncology literature, several studies have examined cyclophosphamide-induced amenorrhea in women receiving traditional IVCY regimens in SLE [5-11]. In an attempt to reduce toxicity of cyclophosphamide, a low-dose IVCY regimen (6 fortnightly pulses at a fixed dose of 500 mg, followed by azathioprine) was proposed on the basis of the results of the Euro-Lupus Nephritis Trial; clinical results comparable to those obtained using the traditional regimen have been reported [12]. However, the incidence of major side effects did not differ significantly between the traditional and the low-dose IVCY regimen groups, perhaps because of the low number of subjects included in the original study [12]. Thus, the effects of low-dose IVCY on cyclophosphamide-induced amenorrhea have not been elucidated.

We conducted a retrospective analysis of the menstrual function of women with SLE before and after receiving low-dose IVCY (500 mg/pulse/month; the duration of treatment was modified depending on clinical response). The objective of this study was to gain a better understanding of the rate of amenorrhea in premenopausal women who were given low-dose IVCY with high-dose glucocorticoid or high-dose glucocorticoid alone. The incidence of the resumption of menses was also assessed. The results of this study will assist physicians in counseling their SLE patients about the benefits and risks of low-dose IVCY and allow women to make better informed decisions. Improved knowledge will also facilitate earlier intervention and treatment of menopausal-related complications.

## Methods

### Patient population

We performed a case-control study of women ≤ 45 years old who were treated for active SLE with either low-dose IVCY (500 mg/body/pulse) plus high-dose glucocorticoids (0.8-1.0 mg/kg/day of prednisolone; IVCY group) or with high-dose glucocorticoids alone (0.8-1.0 mg/kg/day of prednisolone; steroid group) at the Tokyo Women's Medical University hospital from 2000 through 2009. All of these patients had 4 or more revised American College of Rheumatology (ACR) criteria for SLE [13,14]. Patients who had been treated with cyclophosphamide before this study were excluded. One patient with pre-existing amenorrhea and 40 patients who dropped out from our follow-up were excluded. Questionnaires about secondary amenorrhea were sent to the remaining 72 patients. Of these, 32 patients had received low-dose IVCY treatment, and 40 had received high-dose glucocorticoid alone. Medical records from all cases and controls were reviewed. Treatment with glucocorticoids or immunosuppressive drugs was instituted independent of this study. IVCY was primarily administered for SLE patients with lupus nephritis. Other indications for the use of IVCY included central nervous system (CNS) syndrome, autoimmune thrombocytopenia, autoimmune hemolytic anemia, vasculitis and myositis, all of which had been refractory to glucocorticoids. A low-dose IVCY regimen with 500 mg/body/pulse in combination with high-dose glucocorticoids (0.8-1.0 mg/kg/day of prednisolone) was usually used. The duration of IVCY courses was adjusted according to clinical response and adverse effects. None of the women used oral or intramuscular contraceptives during the study period. This study was approved by the Ethical Committee of our institution, and the Helsinki Declaration was followed throughout the study.

### Definition of amenorrhea

Amenorrhea was defined as the cessation of menses for at least 3 consecutive months. Sustained amenorrhea was defined as the cessation of menses for at least 12 consecutive months without resumption during the study period. All patients with amenorrhea followed up for at least additional 12 months to conclude on the sustained, irreversible loss of menstruation. Hormonal studies were not performed to support the diagnosis of ovarian failure in the present study. The average age of Japanese women at menopause is approximately 50 years old according to the Japan Society of Obstetrics and Gynecology.

### Data collection

The information obtained from the medical records of subjects included demographic data (e.g., such as the age at initiation of treatment), the clinical manifestations of SLE, and laboratory data. The activity of SLE was measured using the SLE Activity Index 2000 (SLEDAI-2K) [15]. Other data about subject treatment were also obtained from medical records, including the maximum dose of prednisolone, the number of courses of glucocorticoid pulse therapy, the cumulative dose of cyclophosphamide, the number of pulses of IVCY, and any other immunosuppressants used. Information on the subjects' menstrual history, including development of amenorrhea, duration of amenorrhea, resumption of menses, time to resumption of menses, and other possible causes for amenorrhea was obtained from medical records and returned questionnaires. Informed consent was obtained from all participants.

### Statistical analyses

The incidence of amenorrhea and the association between amenorrhea and clinical parameters were analyzed statistically. Two-group comparisons were analyzed as univariate analyses using Fisher's exact test or a logistic regression analysis for categorical variables and the Mann-Whitney *U *test for continuous variables. Values of *p *< 0.05 were considered statistically significant, and all tests were 2-tailed. The odds ratios (ORs) and their 95% confidence intervals (CIs) as risks for amenorrhea were also calculated. Finally, a stepwise multivariate logistic regression analysis was used to determine the risk factors for amenorrhea. All statistical analyses were performed using JMP statistical software (Ver. 9.0, SAS Institute, Cary, NC, USA).

## Results

### Characteristics of the study subjects

Ultimately, a total of 62 female subjects with SLE provided informed consent for inclusion in this study and returned the questionnaire, representing 86% of the number invited to participate; 29 subjects were treated with IVCY in combination with glucocorticoids (IVCY group), and 33 were treated with glucocorticoids alone (steroid group). The duration of follow-up after initiation of IVCY or high-dose glucocorticoid treatment ranged from 1 to 10 years. The subjects were all Japanese. The characteristics of the subjects are summarized in Table 1. There was no significant difference in age, maximum dose of prednisolone, or the number of subjects treated with glucocorticoid pulse therapy between the groups. Reflecting the indications for IVCY therapy, the SLEDAI-2K score was higher in the IVCY group than in the steroid group (*p *= 0.0004). In the IVCY group, the median cumulative dose of cyclophosphamide was 1000 mg. All subjects in both groups were successfully treated and discharged. Although 3 subjects in the IVCY group and 2 subjects in the steroid group later needed additional oral immunosuppressant (tacrolimus, cyclosporine, or methotrexate), amenorrhea did not develop in any of these subjects during the study period. Azathioprine was not used in the study subjects.

**Table 1:** Characteristics of study subjects with SLE (IVCY group and steroid group)

	IVCY group	Steroid group	*p*^†^
	n = 29	n = 33	
Age (years)	31 (18-45)	30 (19-45)	0.16
Subjects < 40 years old	21 (72%)	30 (91%)	
Subjects ≥ 40 years old	8 (28%)	3 (9%)	0.09
Duration of SLE (months)	7 (1-153)	2 (1-241)	0.05
SLE manifestation			
Nephritis	15 (52%)	13 (39%)	0.44
CNS lupus	7 (24%)	1 (3%)	0.02
Vasculitis	5 (17%)	1 (3%)	0.09
Cytopenia	7 (24%)	10 (30%)	0.78
SLEDAI-2K	16 (2-31)	10 (3-18)	0.0004
Max. dose of prednisolone (mg)	50 (14-125)	45 (40-100)	0.79
Glucocorticoid pulse therapy	6 (20%)	6 (18%)	1.00
Total dose of cyclophosphamide (mg)	1000 (500-6500)		
Total number of IVCY (time)	2 (1-13)		

*Except where indicated otherwise, values of continuous data are the median (range).

^†^*p *values were determined by Fisher's exact test or Mann-Whitney *U *test.

IVCY intravenous cyclophosphamide, SLE systemic lupus erythematosus, CNS central nervous system, SLEDAI-2K systemic lupus erythematosus disease activity index 2000.

### Associations between amenorrhea and clinical parameters

Age at the initiation of treatment did not differ significantly between subjects with and without amenorrhea (*p *= 0.19) (Table 2). Similarly, the logistic regression analysis did not reveal significant correlation between age and amenorrhea (*p *= 0.13; OR 1.06; 95% CI 0.98-1.14). However, when all 62 study subjects were divided into two age groups, those < 40 years old and those ≥ 40 years old at the initiation of treatment, the univariate analyses revealed that the highest risk for developing amenorrhea was being ≥ 40 years old (82 vs. 33%; *p *= 0.005; OR 9.0; 95% CI 1.7-46.4) (Table 2). When the patients were divided into the age groups of 32, 35, or 37 years, there was no or only weaker statistical association (*p *= 0.12, 0.06, and 0.04, respectively). In addition, from the univariate analyses, the incidence of amenorrhea was significantly higher in the IVCY group than in the steroid group (59 vs. 27%; *p *= 0.02; OR 3.8; 95% CI 1.3-11.0), but the incidence of sustained amenorrhea was not (14 vs. 3%; *p *= 0.18). When the total dose of cyclophosphamide was considered as a numerical predictor variable and a univariate logistic regression analysis was applied for all of the patients, including patients from both the IVCY and steroid groups, the OR was 1.0 (*p *= 0.08; 95% CI 1.0-1.0) for amenorrhea. Thus, treatment with IVCY may display a trend to be weakly associated with amenorrhea and sustained amenorrhea. No significant associations between amenorrhea or sustained amenorrhea and any of the other clinical parameters were found in this study (summarized in Table 2).

**Table 2:** Comparisons of clinical parameters of SLE patients with and without amenorrhea

	With amenorrhea	Without amenorrhea	*p*^†^	OR^‡ ^(95% CI)
	n = 26	n = 36		
Age (years)	33 (18-45)	30 (19-44)	0.20	1.0 (1.0-1.1)
Subjects < 40 years old	17 (65%)	34 (94%)	0.005	9.0 (1.7-46.4)
Subjects ≥ 40 years old	9 (35%)	2 (6%)		
Duration of SLE (months)	5 (1-241)	3 (1-128)	0.77	1.0 (1.0-1.0)
SLE manifestation				
Nephritis	12 (46%)	16 (44%)	1.00	1.1 (0.4-3.0)
CNS lupus	5 (19%)	3 (8%)	0.26	2.6 (0.6-12.1)
Vasculitis	2 (8%)	4 (11%)	1.00	0.7 (0.1-3.9)
Cytopenia	8 (31%)	9 (25%)	0.77	1.3 (0.4-4.1)
Positive anti-SS-A/Ro antibodies	14 (54%)	21 (58%)	0.80	0.8 (0.3-2.3)
Positive anti-U1-RNP antibodies	10 (38%)	16 (44%)	0.61	0.7 (0.3-1.9)
SLEDAI-2K	13.5 (5-31)	11.5 (2-26)	0.35	0.9 (0.9-1.0)
Max. dose of prednisolone (mg)	50 (25-125)	45 (14-100)	0.43	1.0 (1.0-1.0)
Glucocorticoid pulse therapy	6 (23%)	6 (17%)	0.54	1.5 (0.4-5.3)
IVCY	17 (65%)	12 (33%)	0.02	3.8 (1.3-11.0)
Total dose of cyclophosphamide (mg)	1300 (500-6500)	1000 (500-2700)	0.69	-
Total number of IVCY (time)	2 (1-13)	2.5 (1-5)	1.00	-

*Except where indicated otherwise, values of continuous data are the median (range). Total dose of IVCY and total number of IVCY were calculated with the subjects in the IVCY group only.

^†^*p *values were determined by Fisher's exact test or Mann-Whitney *U *test.

^‡^ORs of numerical valuables were determined by univariate logistic regression.

IVCY intravenous cyclophosphamide, SLE systemic lupus erythematosus, OR odds ratio, CI confidence interval, CNS central nervous system, SLEDAI-2K systemic lupus erythematosus disease activity index 2000.

### Incidence of amenorrhea in SLE subjects by age and treatment groups

Amenorrhea developed more frequently in subjects < 40 years old in the IVCY group than in the steroid group (11/21 vs. 6/30; *p *= 0.03; OR 4.4; 95% CI 1.3-15.2) (Table 3). Subjects ≥ 40 years old had a high incidence of amenorrhea in both treatment groups with no statistical difference between the groups (6/8 in the IVCY group; 3/3 in the steroid group, *p *= 1.00) (Table 3).

**Table 3:** Incidence of amenorrhea in SLE patients in each age and treatment group

	With amenorrhea	Without amenorrhea	p*	OR (95% CI)
	n = 26	n = 36		
Age < 40 years old				
IVCY group^†^	11 (42%)	10 (28%)	0.03	4.4 (1.3-15.2)
Steroid group^†^	6 (23%)	24 (67%)	-	-
Age ≥ 40 years old				
IVCY group^†^	6 (23%)	2 (6%)	1.00	-
Steroid group^†^	3 (12%)	0 (0%)	-	-

*p values were determined by Fisher's exact test.

^†^Patients with SLE were divided into 2 groups: those who received low-dose IVCY plus high-dose glucocorticoids (IVCY group) and those who received high-dose glucocorticoids alone (steroid group).

IVCY intravenous cyclophosphamide, SLE systemic lupus erythematosus, OR odds ratio, CI confidence interval.

### Frequency of resumption of menses

There was no statistical difference in the frequency of resumption of menses between the IVCY group and the steroid group (13/17 and 8/9, respectively, *p *= 0.63) (Table 4). In the patients with transient amenorrhea, time to resumption of menses was 8 (3-18) months in the IVCY group and 6 (3-12) months in the steroid group, respectively (median and range, respectively): there was no statistical difference (*p *= 0.91). The logistic regression analysis revealed a significant correlation between age and sustained amenorrhea (*p *< 0.0001). Sustained amenorrhea developed in 4 subjects in the IVCY group and in 1 subject in the steroid group; all of these subjects were > 40 years old. Conversely, menses resumed in all subjects < 40 years old in both treatment groups. Thus, menses resumed more frequently in subjects < 40 years old than in the subjects > 40 years old (*p *= 0.002) (Table 4). Unfortunately, the small sample size of our study hampered the power to statistically evaluate any significant association of IVCY with sustained amenorrhea in the both age groups.

**Table 4:** Comparisons of menses resumption by age or treatment regimen groups

	Resumed menses	Sustained amenorrhea	p*	OR^† ^(95% CI)
	n = 21	n = 5		
Treatment regimen				
IVCY group^‡^	13 (62%)	4 (80%)	0.63	2.5 (0.2-26.1)
Steroid group^‡^	8 (38%)	1 (20%)		-
Age				
< 40 years old	17 (81%)	0 (0%)	0.002	-
≥ 40 years old	4 (19%)	5 (100%)		-
Age < 40 years old				
IVCY group^‡^	11 (52%)	0 (0%)	1.00	-
Steroid group^‡^	6 (29%)	0 (0%)		-
Age ≥ 40 years-old				
IVCY group^‡^	2 (10%)	4 (80%)	0.52	4.0 (0.2-75.7)
Steroid group^‡^	2 (10%)	1 (20%)		-

*p values were determined by Fisher's exact test.

^†^ORs were defined as the ratios of the odds of sustained amenorrhea.

^‡^Patients with SLE were divided into 2 groups: those who received low-dose IVCY plus high-dose glucocorticoids (IVCY group) and those who received high-dose glucocorticoids alone (steroid group).

IVCY intravenous cyclophosphamide, SLE systemic lupus erythematosus, OR odds ratio, CI confidence interval.

### Multivariate analysis of risk for amenorrhea

Using a stepwise multiple regression analysis, a significant association between amenorrhea and being ≥ 40 years old was found (*p *= 0.009; OR 10.2; 95% CI 1.8-58.7) (Table 5). Treatment with IVCY may show a trend to be weakly associated with amenorrhea (*p *= 0.07; OR 2.9; 95% CI 0.9-9.4). In addition, when the total dose of cyclophosphamide was considered as a numerical predictor variable, and a similar multivariate logistic regression analysis was applied for all of the patients, including patients from both the IVCY and steroid groups, the OR of IVCY was 1.0 (95% CI 1.0-1.0; *p *= 0.11) for amenorrhea. The multivariate analysis of sustained amenorrhea was not performed because the overall low incidence yielded insufficient numbers for analysis.

**Table 5:** Multivariate analysis of associations between clinical parameters and amenorrhea

Clinical parameter	p*	OR* (95% CI)
Age ≥ 40 years old	0.009	10.2 (1.8 - 58.7)
IVCY	0.07	2.9 (0.9 - 9.4)
Maximum dose of prednisolone	0.10	1.0 (1.0 - 1.1)

*p values and ORs were determined by stepwise multivariate logistic regression using a model adjusted for the following variables: 'Age ≥ 40 years-old'; IVCY; maximum dose of prednisolone; steroid pulse therapy; SLEDAI-2K.

IVCY intravenous cyclophosphamide, SLE systemic lupus erythematosus, OR odds ratio, CI confidence interval.

### Other adverse events

Major potentially IVCY-related adverse events in the study subjects are summarized in Table 6. The incidence of adverse events other than amenorrhea was low, and no significant differences between the IVCY group and the steroid group were found.

**Table 6:** Major adverse events in study subjects with SLE

Adverse events*	IVCY group	Steroid group
	n = 29	n = 33
Amenorrhea	17 (59%)	9 (27%)
Elevation of liver enzymes	2 (7%)	0 (0%)
Hematological disorder	1 (3%)	0 (0%)
Herpes Zoster infection	1 (3%)	2 (6%)
Cytomegalovirus infection	0 (0%)	1 (3%)
Tinea corporis	0 (0%)	1 (3%)
Myocarditis	1 (3%)	0 (0%)

*Adverse events that are usually not regarded as IVCY-related are excluded from this table.

IVCY intravenous cyclophosphamide, SLE systemic lupus erythematosus.

## Discussion

The important findings of this study are that i) patients < 40 years old have a minimum risk for sustained amenorrhea with low-dose IVCY treatment; ii) irrespective of treatment regimen, sustained amenorrhea developed frequently in patients ≥ 40 years old at initiation of treatment; iii) even low-dose IVCY may increase the risk of transient amenorrhea; and iv) the strongest risk factor for developing amenorrhea in SLE patients treated with high-dose glucocorticoids with or without low-dose IVCY is being ≥ 40 years old at the initiation of treatment.

The low-dose IVCY treatment regimen was initially proposed as an attempt to reduce the toxicity of cyclophosphamide [12]. However, the incidence of major side effects, including menopause, did not differ significantly between traditional and low-dose IVCY regimen groups (menopause; 2/45 and 2/44, respectively) in the original study [12]. Although patients treated with high-dose or traditional IVCY regimens were not included in the present study, the proportion of sustained amenorrhea in patients < 40 years old in our study (0/51) was much lower than similar figures for patients treated with the traditional IVCY regimen in other studies. At initiation of traditional IVCY therapy, Boumpas et al. reported that sustained amenorrhea developed in 19% (6/31) of women ≤ 30 years old [5], Mok et al. reported that sustained amenorrhea developed in 19% (10/54) of women < 40 years old [7], Park et al. reported that sustained amenorrhea developed in 11% (7/62) of women ≤ 40 years old [8], and Ioannidis et al. reported that sustained amenorrhea developed in 11% (5/44) of women ≤ 31 years old [11]. Thus, when considering the benefits of low-dose IVCY and the risk for amenorrhea, treatment with low-dose IVCY would have a lower risk for sustained amenorrhea than higher-dose regimens and should be well tolerated in premenopausal patients < 40 years old. In addition, previous reports demonstrate that the rates of amenorrhea following short-term high-dose IVCY are also lower than those following long-term high-dose IVCY: 0-12% [5,8,10]. Because our IVCY protocol in the present study was mostly both low-dose and short-term, the duration of IVCY treatment and cumulative dose of cyclophosphamide also affect the resumption of menses. However, our study shows that even low-dose IVCY may increase the risk of transient amenorrhea, an important clinical consideration that should not be overlooked.

Consistent with previous studies using the traditional IVCY regimen [5,7,8,11] and with breast cancer studies [4,16], sustained amenorrhea developed frequently in patients ≥ 40 years old at initiation of treatment, irrespective of treatment regimen. The strongest risk factor for developing amenorrhea in SLE patients treated with high-dose glucocorticoids with or without low-dose IVCY was being ≥ 40 years old at the initiation of treatment in the present study. Older women are more likely to progress to premature ovarian failure after therapy because they have a smaller number of oocytes at the initiation of treatment [17]. Thus, the higher risk of sustained amenorrhea from IVCY therapy should be a serious consideration in patients > 40 years old. In addition, when the patients were divided into the age groups of 32, 35, or 37 years, there was no or only weaker statistical association, although there might be an increasing risk for amenorrhea after the age of 35 years. Age at the initiation of treatment did not differ significantly between subjects with and without amenorrhea, and the logistic regression analysis did not reveal significant correlation between age and amenorrhea. Thus, we speculate that the age of 40 was a threshold for the patients in the present study.

Cyclophosphamide toxicity in women may arise from direct effects on the oocyte, or indirectly through its effects on the supporting granulosa cells of the follicle through gap junctions connecting these "nursing cells" to the oocyte [17]. DNA cross-linking occurs in granulosa cells of animals treated with cyclophosphamide within 2 hours of administration, as well as increased nuclear size, which is thought to result from a cell-cycle block in G_2 _phase [17]. Following exposure to cyclophosphamide, human ovaries are fibrosed, and follicles are destroyed [17]. Ovarian dysfunction can be partially mitigated during IVCY therapy by using gonadotrophic-releasing hormone analogues, which reduce proliferation within the ovary [17]. However, the risk of precipitating a disease flare with hormonal stimulation must be considered when treating SLE [17].

One of the limitations of the present study is the selection bias inherent in a single-center retrospective study, although rarity of the disease makes performing prospective studies difficult. In addition, our subject number was insufficient for definitive conclusions about association between amenorrhea and low-dose IVCY, and we lacked controls such as age-matched healthy subjects or SLE patients who were not treated with glucocorticoids. However, the size of our study population is comparable to similar previously reported studies, and treatment without glucocorticoids in active SLE is impractical. Another potential weakness arises from the lack of uniformity of the follow-up duration and treatment regimens, although all study subjects were followed up for at least 1 year, usually sufficient time to evaluate drug-induced amenorrhea and menses resumption. Evaluation of ovarian function could not be included because we did not measure the hormone levels of all of the subjects. The fact that the SLEDAI-2K score was higher in the IVCY group compared with the steroid group may be a limitation of this study because high disease activity has previously been associated with the development of amenorrhea in SLE [18]. However, in the present study, there was no association between the SLEDAI-2K score and amenorrhea. Finally, because this study was not designed to assess the effectiveness of low-dose IVCY, it is not clear whether our low-dose IVCY regimen is applicable to severe manifestations of SLE. Considering that the patients that could not respond to the questionnaire due to severe or fatal disease were excluded from the study and that some of the study subjects required additional therapy, our low-dose IVCY regimen would not necessarily be suitable for all severe manifestations of SLE.

In conclusion, SLE patients < 40 years old have a minimum risk for sustained amenorrhea with low-dose IVCY treatment, whereas higher-dose IVCY reportedly has a higher risk even in this younger population. In contrast, sustained amenorrhea developed frequently in patients ≥ 40 years old at the initiation of treatment; this higher risk of sustained amenorrhea with IVCY therapy requires serious consideration when treating SLE patients ≥ 40 years old. However, our study demonstrates the possibility that even low-dose IVCY may increase the risk of amenorrhea, an important clinical consideration that should not be overlooked.

## Conclusions

Although low-dose IVCY may increase the risk for amenorrhea, our data suggest that patients < 40 years old have a minimum risk for sustained amenorrhea with low-dose IVCY treatment. A higher risk for sustained amenorrhea following treatment with IVCY is a consideration for patients ≥ 40 years old.

## Competing interests

The authors declare that they have no competing interests.

## Authors' contributions

SB and YKatsumata conceived the study and drafted the manuscript. YKawaguchi, TG, TS and TK participated in acquisition of data and patient recruitment. MH and HY participated in the design and coordination of the study. All authors read and approved the final manuscript.

## Pre-publication history

The pre-publication history for this paper can be accessed here:

http://www.biomedcentral.com/1472-6874/11/28/prepub
